# Prevalence of poor sleep quality and its associated factors in nurses: a multicenter cross-sectional study

**DOI:** 10.3389/fpubh.2026.1927513

**Published:** 2026-08-07

**Authors:** Min Li, Xiao-Feng Zheng, Xiu-Chun Yin, Qian-Qian Mou

**Affiliations:** 1 Department of General Surgery, Division of Vascular Surgery, West China Hospital, Sichuan University, Chengdu, China; 2 Department of Radiology, West China Hospital, Sichuan University, Chengdu, China; 3 Clinical Trial Center, West China School of Nursing, West China Hospital, Sichuan University, Chengdu, China

**Keywords:** nurses, prevalence, self-regulatory fatigue, sleep quality, workload

## Abstract

**Background:**

Although poor sleep quality has been significantly associated with burnout, medical errors, and patient safety, its prevalence and associated factors among nurses remain underexplored. This study aimed to determine the prevalence of poor sleep quality and identify its associated factors in nurses.

**Methods:**

In this multicenter cross-sectional study, convenience sampling was employed to enroll nurses from eight tertiary hospitals across Sichuan Province, China, between October and December 2025. Participants completed a demographic questionnaire, the Pittsburgh Sleep Quality Index, the NASA Task Load Index, and the Self-Regulatory Fatigue Scale. Univariate analysis and binary logistic regression were used to examine the correlates of poor sleep quality.

**Results:**

A total of 1,289 nurses were included, with 813 (63.1%) reporting poor sleep quality. Age (31–45 years: OR = 1.628, 95% CI: 1.200–2.209, *p* = 0.002; 46–60 years: OR = 2.440, 95% CI: 1.608–3.702, *p* < 0.001), work unit (pediatric ward: OR = 2.063, 95% CI: 1.407–3.024, *p* < 0.001; intensive care unit: OR = 3.178, 95% CI: 1.993–5.068, *p* < 0.001; emergency department: OR = 2.492, 95% CI: 1.664–3.732, *p* < 0.001), work experience (11–20 years: OR = 1.430, 95% CI: 1.031–1.984, *p* = 0.032; > 20 years: OR = 2.237, 95% CI: 1.422–3.518, *p* < 0.001), night shift (OR = 1.857, 95% CI: 1.381–2.498, *p* < 0.001), workload (OR = 1.223, 95% CI: 1.142–1.261, *p* < 0.001), and self-regulatory fatigue (OR = 1.346, 95% CI: 1.221–1.384, *p* < 0.001) were identified as significantly associated factors of poor sleep quality in nurses.

**Conclusion:**

Poor sleep quality was highly prevalent among nurses and was significantly associated with age, work unit, work experience, night shift, workload, and self-regulatory fatigue. These findings highlight the importance of prioritizing high-risk populations, particularly older nurses and those working in high-acuity settings, with lengthy service, and on rotating night schedules. Moreover, comprehensive strategies addressing workplace stressors and individual resilience may help reduce sleep problems.

## 1 Introduction

Adequate sleep constitutes a basic physiological necessity for human beings (1). In the field of occupational health, good sleep quality is critical to preserving employees’ holistic health (2). However, poor sleep quality has become an increasingly prominent issue among healthcare professionals, especially nurses, who routinely face high-pressure working conditions marked by extended duty hours, high patient-to-nurse ratios, and intense emotional labor (3, 4). A meta-analytic estimate indicated a global prevalence of 61.0% for poor sleep quality among nurses (5). A growing body of literature has indicated that poor sleep quality exerts deleterious effects on nurses’ physical and psychological well-being, manifesting as elevated risks of burnout, depressive symptoms, and cardiovascular morbidity (6, 7). Meanwhile, poor sleep quality predisposes to medication errors and poses a direct threat to patient safety (8). The burden of poor sleep quality among nurses remains substantial despite heightened awareness of this occupational health concern. Therefore, identification of factors associated with poor sleep quality in nurses is imperative for the development of evidence-based preventive strategies and targeted interventions to improve their sleep health.

Studies conducted among nurses have identified several potential factors associated with poor sleep quality (9–12). In terms of demographic characteristics, age, marital status, work unit, work experience, and night shift work have all been demonstrated to be significantly correlated with poor sleep quality in nurses (9–12). Based on the Stress–Strain Model, chronic exposure to occupational stressors without adequate recovery leads to the progressive accumulation of strain, ultimately manifesting as adverse health outcomes, including sleep impairment (13). Within this theoretical framework, workload could be conceptualized as a core external stressor in the nursing profession. Nurses generally contend with heavy workload and intense emotional demands in daily clinical practice, placing them in a chronically high-stress environment (14). Workload not only prolongs the duration of physical and mental exertion during shifts but also intrudes into off-duty time through cognitive rumination, thereby impeding the psychological detachment and resource recovery essential for restorative sleep (15). Self-regulatory fatigue is regarded as a state of depletion of internal psychological resources (16). According to the Conservation of Resources Theory, individuals strive to obtain and safeguard their psychological resources (17). Perceived threats of resource depletion or unsuccessful resource investment with insufficient returns give rise to psychological strain (18). In the nursing context, the sustained engagement in heavy emotional labor may gradually deplete these resources, rendering nurses more susceptible to pre-sleep anxiety and diminished cognitive control, which in turn may contribute to poor sleep quality (19). Taken together, the Stress–Strain Model and the Conservation of Resources Theory converge to suggest a dual-pathway mechanism through which workload (as an external stressor) and self-regulatory fatigue (as a manifestation of internal resource depletion) may independently and interactively contribute to poor sleep quality among nurses. We therefore hypothesized that higher levels of workload (H1) and higher levels of self-regulatory fatigue (H2) would each be significantly associated with an increased risk of poor sleep quality among nurses. Despite these established associations, the effects of workload and self-regulatory fatigue on poor sleep quality in nurses remain underexplored, particularly across diverse clinical settings. Specifically, prior investigations have predominantly concentrated on intensive care units and emergency departments (9, 10), while general medical-surgical wards, psychiatric units, operating rooms, and outpatient departments have received considerably less empirical attention despite their distinct workload characteristics and emotional demands. This uneven distribution of evidence limits the comprehensiveness of our understanding regarding how occupational factors affect sleep quality across the full spectrum of nursing practice. Therefore, this multicenter cross-sectional study was conducted to investigate the prevalence of poor sleep quality and its associated factors among nurses, with particular emphasis on the roles of workload and self-regulatory fatigue, thereby providing an empirical basis for developing targeted multilevel interventions.

## 2 Methods

### 2.1 Study design, setting, and participants

A multicenter cross-sectional design was adopted, utilizing convenience sampling to recruit clinical nurses from eight tertiary hospitals across Sichuan Province, China, between October and December 2025. Eligible participants were required to hold a valid nursing practice certificate, be employed in an unique department, and provide direct clinical care to patients. Nurses who were pregnant, on sick leave, or on personal leave during the survey period, as well as those in administrative or managerial positions without direct patient care duties, were excluded from the study.

*A priori* sample size calculation was performed using PASS software. According to a meta-analysis which reported that the pooled prevalence of poor sleep quality in nurses was 61.0% (5), we adopted the following parameters for *a priori* sample size calculation: a 95% confidence level, an anticipated proportion of 0.61, and a two-sided confidence interval width of 0.10. Accounting for an anticipated 15% invalid response rate, the final target sample size was set at 431 participants. However, given the multicenter design of this study and the anticipated variability in response rates across different hospitals, we aimed to recruit a larger sample to ensure sufficient statistical power for subgroup analyses by clinical department. In addition, the use of a WeChat-based QR code distribution platform enabled broad and efficient dissemination, which naturally led to a higher participation rate than initially expected. Ultimately, 1,289 valid questionnaires were collected. This oversampled size not only meets the minimum requirement but also improves the precision of estimates and enhances the representativeness of the study population across diverse clinical settings.

### 2.2 Measurements

#### 2.2.1 Demographic information form

The demographic information form included eight variables, including gender, age, marital status, educational level, work unit, work experience, monthly income (CNY), and night shift.

#### 2.2.2 Pittsburgh sleep quality index

Sleep quality was evaluated using the Chinese version of the PSQI (20), a widely-used 19-item instrument that assesses seven clinically-relevant dimensions of sleep: subjective quality, latency, duration, efficiency, disturbances, medication use, and daytime dysfunction. Each item is rated on a 4-point scale from 0 (no difficulty) to 3 (severe difficulty), yielding a global score ranging from 0 to 21, with higher values indicating worse sleep. Consistent with prior research, a global PSQI score >5 was used to identify poor sleep quality. The Cronbach’s *α* for the PSQI in our sample was 0.826, indicating good internal consistency.

#### 2.2.3 National aeronautics and space administration task load index

Workload was measured using the Chinese version of the NASA-TLX (21). The scale comprises six items: mental demand, physical demand, temporal demand, performance, effort, and frustration. Each item is rated from 0 (very low) to 20 (very high), and the total score (0–120) is obtained by summing all items, with higher scores indicating higher workload. In this study, the instrument demonstrated good reliability, with a Cronbach’s *α* of 0.817.

#### 2.2.4 Self-regulatory fatigue scale

To assess self-regulatory fatigue, we employed the Chinese version of the SRF-S (22), a 16-item self-report scale across three dimensions: cognitive regulation, emotional regulation, and behavioral regulation. Responses are recorded on a 5-point Likert scale (1 = strongly disagree, 5 = strongly agree), and the sum of all item scores yields a global score ranging from 16 to 80, with higher scores denoting greater self-regulatory fatigue. The internal consistency of the SRF-S in our study was robust (Cronbach’s *α* = 0.811).

### 2.3 Data collection

**Figure 1:**
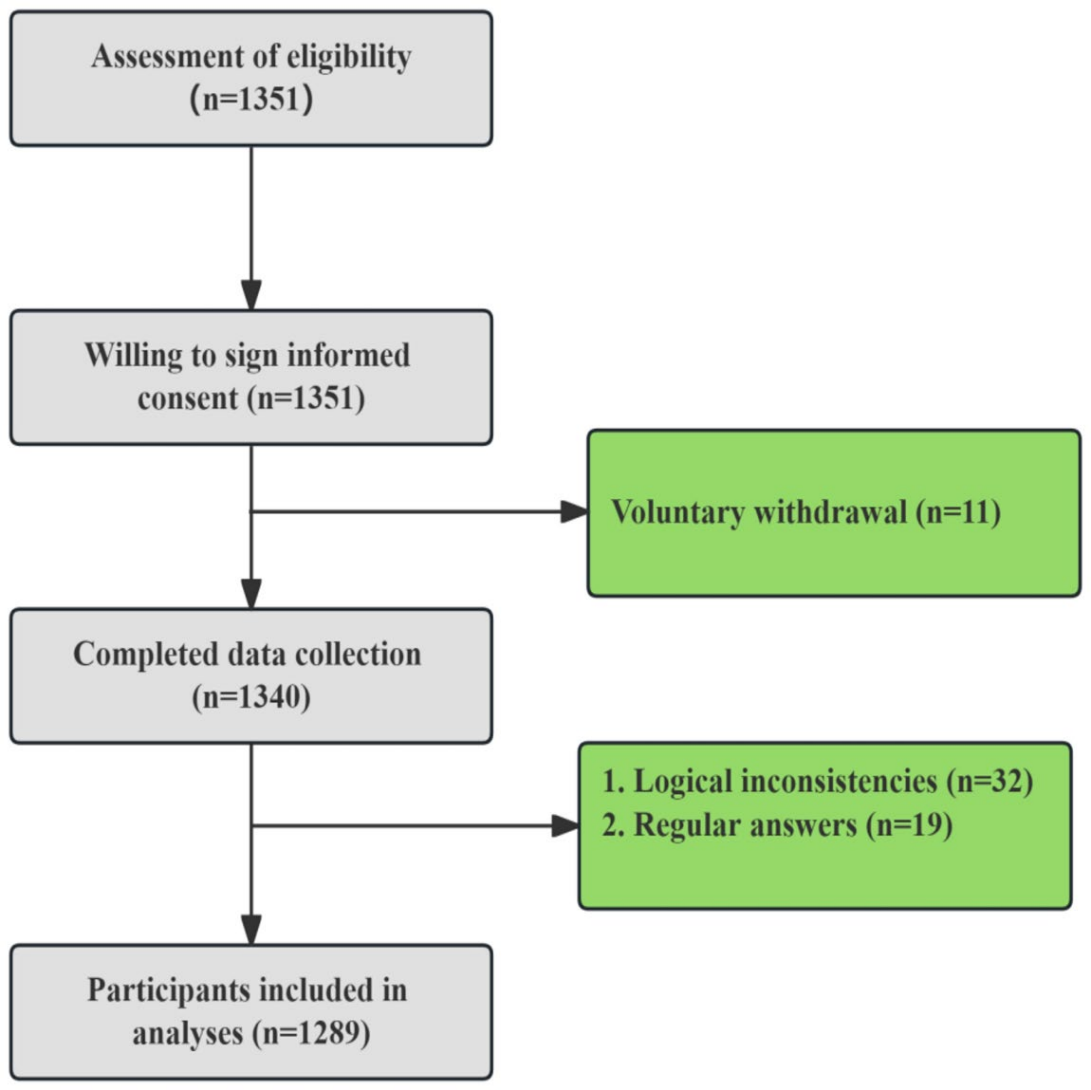
Enrollment diagram of the participant selection process.

Data collection was conducted using the Questionnaire Star platform. We collaborated with the nursing management departments of the participating hospitals. To enhance sample diversity and reduce self-selection bias, nursing manager distributed the QR code of the online questionnaire to eligible clinical nurses via WeChat. The recruitment notice clearly described the purpose, significance, and instructions for questionnaire completion, and emphasized that participation was entirely voluntary, with the assurance that neither refusal nor withdrawal would affect their performance appraisals. All participants provided electronic informed consent before completing the questionnaire. To ensure data quality, we restricted each IP address to a single submission, required responses to all items, and screened the anonymized data for invalid patterns. After data collection, we screened the anonymized data for invalid response patterns using predefined criteria: (1) logical inconsistencies were defined as contradictory answers to paired items, such as reporting “never” experiencing a specific sleep-related symptom while simultaneously endorsing a frequency of “three or more times per week” for the same symptom on corresponding items within the Pittsburgh Sleep Quality Index; (2) regular responses were defined as identical answer choices across more than 90% of consecutive items, or a clearly patterned sequence of responses. A total of 1,351 eligible nurses were identified, and 1,340 of them completed the survey. After excluding invalid responses, 1,289 valid questionnaires were included, representing a valid response rate of 95.4%. The flow diagram of participant enrollment is provided in Figure 1.

### 2.4 Statistical analysis

Statistical analysis was performed using SPSS 27.0. Continuous data were presented as means and standard deviations, and categorical data as frequencies and percentages. Between-group differences were examined using independent-samples *t*-tests and chi-square tests to compare nurses with good and poor sleep quality. Moreover, a binary logistic regression was conducted to identify factors associated with poor sleep quality. Multicollinearity among the independent variables was assessed using the variance inflation factor (VIF), with a threshold of >5 indicating significant collinearity. Model calibration was evaluated using the Hosmer-Lemeshow goodness-of-fit test. *p* < 0.05 was considered statistically significant (two-sided).

### 2.5 Ethical considerations

This study was approved by the Ethics Committee of West China Hospital, Sichuan University (20231846) and followed the Declaration of Helsinki. All participants provided informed consent, and their data remained confidential. To ensure data privacy and anonymity, all responses were collected and stored on the Questionnaire Star platform with encrypted data transmission. No personally identifiable information was collected. The data were accessible only to the research team and were used solely for this study.

## 3 Results

### 3.1 Demographic characteristics of participants

A total of 1,289 nurses were included in the data analysis. The sample was mainly female (95.3%), with the largest age group being 31–45 years (50.0%). Most participants were married (57.5%) and held a bachelor’s degree (57.8%). Monthly income was concentrated in the 5,000–10,000 CNY range (51.9%), while the majority had 1–10 years of work experience (45.7%). In terms of work units, general wards accounted for the largest proportion (45.7%), followed by emergency departments (22.0%), pediatric wards (19.3%), and intensive care units (13.1%). Night shifts were reported by 68.1% of the nurses. The demographic characteristics of participants are detailed in Table 1.

**Table 1:** Demographic characteristics of participants and univariate analysis results.

Variables	Total sample (*n* = 1,289)	Good sleep quality (*n* = 476)	Poor sleep quality (*n* = 813)	*P*
Gender				0.312
Male	61 (4.7%)	26 (5.5%)	35 (4.3%)	
Female	1,228 (95.3%)	450 (94.5%)	778 (95.7%)	
Age (years)				<0.001
18–30	452 (35.1%)	206 (43.3%)	246 (30.3%)	
31–45	645 (50.0%)	216 (45.4%)	429 (52.8%)	
46–60	192 (14.9%)	54 (11.3%)	138 (17.0%)	
Marital status				0.214
Unmarried	469 (36.4%)	181 (38.0%)	288 (35.4%)	
Married	741 (57.5%)	262 (55.0%)	479 (58.9%)	
Divorced/widowed	79 (6.1%)	33 (6.9%)	46 (5.7%)	
Educational level				0.673
Junior college	375 (29.1%)	135 (28.4%)	240 (29.5%)	
Undergraduate	745 (57.8%)	281 (59.0%)	464 (57.1%)	
Postgraduate	169 (13.1%)	60 (12.6%)	109 (13.4%)	
Work unit				<0.001
General wards	589 (45.7%)	284 (59.7%)	305 (37.5%)	
Pediatric ward	248 (19.2%)	68 (14.3%)	180 (22.1%)	
Intensive care unit	169 (13.1%)	28 (5.9%)	141 (17.3%)	
Emergency department	283 (22.0%)	96 (20.2%)	187 (23.0%)	
Work experience (years)				<0.001
1–10	589 (45.7%)	260 (54.6%)	329 (40.5%)	
11–20	505 (39.2%)	168 (35.3%)	337 (41.5%)	
>20	195 (15.1%)	48 (10.1%)	147 (18.1%)	
Monthly income (CNY)				0.873
<5,000	397 (30.8%)	148 (31.1%)	249 (30.6%)	
5,000–10,000	669 (51.9%)	244 (51.3%)	425 (52.3%)	
>10,000	223 (17.3%)	84 (17.6%)	139 (17.1%)	
Night shift				<0.001
No	411 (31.9%)	192 (40.3%)	219 (26.9%)	
Yes	878 (68.1%)	284 (59.7%)	594 (73.1%)	
Workload	81.65 ± 14.28	72.53 ± 12.16	86.94 ± 13.57	<0.001
Self-regulatory fatigue	56.38 ± 9.74	48.62 ± 8.53	60.87 ± 9.12	<0.001

Continuous variables were compared using the independent *t*-test; Categorical variables were compared using the chi-square test.

### 3.2 Prevalence of poor sleep quality in nurses

**Figure 2:**
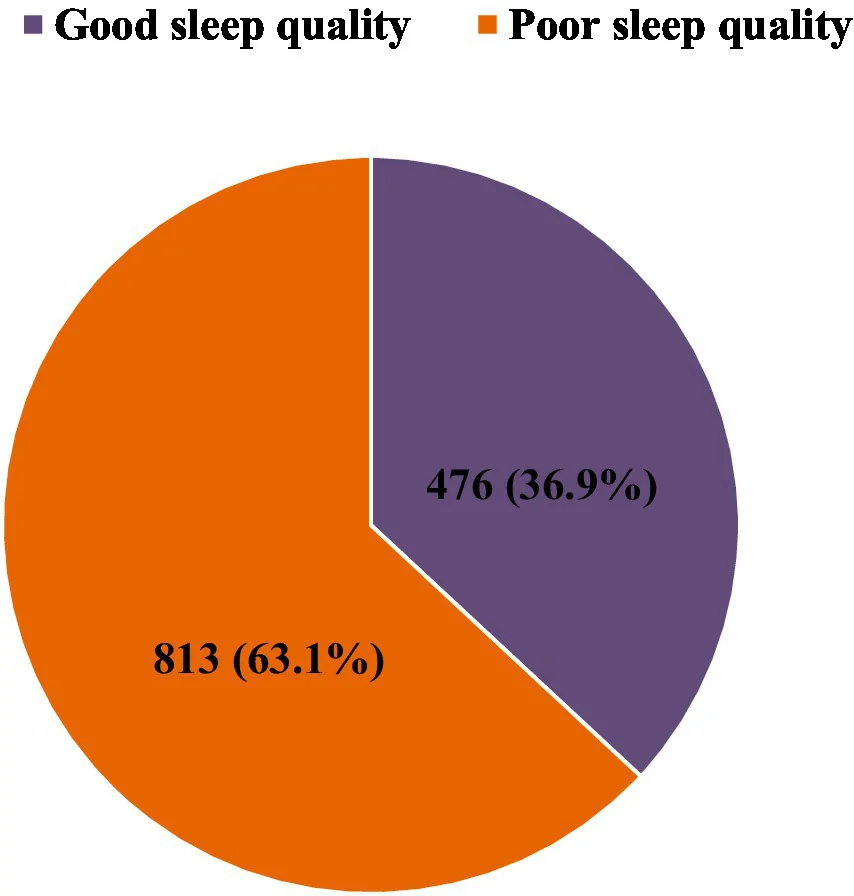
Prevalence of poor sleep quality in nurses.

Of the 1,289 nurses included in the final analysis, 813 were identified as having poor sleep quality, yielding a prevalence rate of 63.1%. The prevalence of poor sleep quality in nurses is illustrated in Figure 2.

### 3.3 Univariate analysis of poor sleep quality in nurses

Univariate analysis was conducted to compare the demographic characteristics of nurses with poor versus good sleep quality (Table 1). Significant differences were observed between the two groups in age, work unit, work experience, night shift, workload, and self-regulatory fatigue (all *p* < 0.001), whereas no significant differences were found in gender (*p* = 0.312), marital status (*p* = 0.214), educational level (*p* = 0.673), and monthly income (*p* = 0.873).

### 3.4 Binary logistic regression analysis of poor sleep quality in nurses

Participants were classified into poor sleep quality (PSQI >5) and good sleep quality (PSQI ≤5) groups according to their total PSQI scores. Binary logistic regression was conducted to explore the associated factors of poor sleep quality in nurses (Table 2). Variables with *p* < 0.05 in univariate analysis (age, work unit, work experience, night shift, workload, and self-regulatory fatigue) were entered as independent variables. Categorical variables were dummy-coded, with the first category as the reference. The variable assignment is presented in Table 3. Age (31–45 years: OR = 1.628, 95% CI: 1.200–2.209, *p* = 0.002; 46–60 years: OR = 2.440, 95% CI: 1.608–3.702, *p* < 0.001), work unit (pediatric ward: OR = 2.063, 95% CI: 1.407–3.024, *p* < 0.001; intensive care unit: OR = 3.178, 95% CI: 1.993–5.068, *p* < 0.001; emergency department: OR = 2.492, 95% CI: 1.664–3.732, *p* < 0.001), work experience (11–20 years: OR = 1.430, 95% CI: 1.031–1.984, *p* = 0.032; > 20 years: OR = 2.237, 95% CI: 1.422–3.518, *p* < 0.001), night shift (OR = 1.857, 95% CI: 1.381–2.498, *p* < 0.001), workload (OR = 1.223, 95% CI: 1.142–1.261, *p* < 0.001), and self-regulatory fatigue (OR = 1.346, 95% CI: 1.221–1.384, *p* < 0.001) were identified as significantly associated factors of poor sleep quality in nurses. Multicollinearity diagnostics showed that VIF values for all independent variables ranged from 1.865 to 3.226, indicating the absence of significant collinearity. The Hosmer-Lemeshow test demonstrated good model fit, with a χ^2^ of 8.24 (*p* = 0.412).

**Table 2:** Binary logistic regression analysis of poor sleep quality in nurses.

Variables	*β*	SE	Wald	*P*	OR	95% CI	VIF
Constant	−4.856	0.623	60.75	<0.001	0.008	–	–
Age (years)							3.226
18–30	Reference						
31–45	0.487	0.156	9.75	0.002	1.628	1.200–2.209	
46–60	0.892	0.213	17.54	<0.001	2.440	1.608–3.702	
Work unit							2.411
General wards	Reference						
Pediatric ward	0.724	0.195	13.79	<0.001	2.063	1.407–3.024	
Intensive care unit	1.156	0.238	23.59	<0.001	3.178	1.993–5.068	
Emergency department	0.913	0.206	19.64	<0.001	2.492	1.664–3.732	
Work experience (years)							1.865
1–10	Reference						
11–20	0.358	0.167	4.60	0.032	1.430	1.031–1.984	
>20	0.805	0.231	12.14	<0.001	2.237	1.422–3.518	
Night shift							2.332
No	Reference						
Yes	0.619	0.151	16.81	<0.001	1.857	1.381–2.498	
Workload	0.182	0.025	52.99	<0.001	1.223	1.142–1.261	2.354
Self-regulatory fatigue	0.262	0.032	67.04	<0.001	1.346	1.221–1.384	3.142

**Table 3:** Binary logistic regression analysis variable assignment.

Variables	Assignment
Dependent variable	
Sleep quality	Good sleep quality = 0, Poor sleep quality = 1
Independent variable	
Age (years)	18–30 = 1, 31–45 = 2, 46–60 = 3
Work unit	General wards = 1, Pediatric ward = 2, Intensive care unit = 3, Emergency department = 4
Work experience	1–10 = 1, 11–20 = 2, >20 = 3
Night shift	No = 0, Yes = 1
Workload	Continuous (total score)
Self-regulatory fatigue	Continuous (total score)

## 4 Discussion

This study aimed to determine the prevalence of poor sleep quality and its associated factors in nurses. The results demonstrated that 63.1% of nurses reported poor sleep quality, which was significantly associated with age, work unit, work experience, night shift, workload, and self-regulatory fatigue. These findings provide an empirical basis for the development of comprehensive organizational management strategies at both the institutional and individual levels, with the goal of effectively reducing the prevalence of poor sleep quality among nurses and fostering a supportive clinical work climate that promotes healthy sleep practices.

Our study revealed that 63.1% of nurses reported poor sleep quality, which is in line with the results reported in earlier investigations (10, 11). The high prevalence of poor sleep quality among nurses can be attributed to the interplay of physiological, occupational, and psychological factors. Frequent night shifts and rotating schedules disrupt circadian rhythms and melatonin secretion, thereby contributing to chronic sleep disturbances (23). Meanwhile, heavy workloads and understaffing impose sustained physical exhaustion and high cognitive demands, leaving insufficient recovery time between shifts (24). In clinical practice, nurses are routinely required to engage in complex symptom management, delivery of unfavorable news, end-of-life discussions, and bereavement support, and these duties consume substantial psychological resources. Over time, repeated exposure to patients’ suffering and existential distress progressively depletes psychological resilience, which in turn further compromises sleep quality (25).

Our findings indicated that age, work unit, work experience, and night shift were significantly correlated with poor sleep quality in nurses. Specifically, nurses who were older, worked in pediatric wards, ICUs, or emergency departments, had longer work experience, or engaged in night shift rotations were at a higher risk of experiencing poor sleep quality. Several factors may account for the observed associations between these variables and poor sleep quality. First, as older nurses tend to experience declines in melatonin secretion and increased sleep fragmentation, rendering them inherently more vulnerable to sleep disturbances, a vulnerability that may be compounded by cumulative career stress and evolving family responsibilities over time (26). Moreover, nurses in pediatric wards, ICUs, and emergency departments operate in high-intensity, unpredictable settings marked by life-threatening emergencies. Such environments induce sustained hyperarousal and, coupled with high patient acuity, rapid turnover, and emotionally intense interactions, afford little opportunity for physical and mental recovery either during or between shifts (27). Although these high-acuity settings share common features, their operational demands differ. Emergency department nurses face unpredictability and extreme case variability, imposing high cognitive load that hinders psychological detachment from work and promotes pre-sleep rumination. In contrast, ICU nurses endure continuous monitoring of critically ill patients and frequent mortality exposure, bearing the burden of prolonged attention and emotional toll, which may lead to cumulative emotional exhaustion (15). Third, longer work experience may paradoxically increase risk through the gradual accumulation of occupational stress, emotional exhaustion. Senior nurses, in particular, often assume additional supervisory responsibilities, which may prolong working hours and heighten cognitive load (28). Finally, night shift work directly undermines sleep physiology by disrupting circadian rhythms and suppressing melatonin production, resulting in both sleep initiation and maintenance difficulties. The problem is exacerbated by irregular scheduling and abbreviated recovery intervals between night shifts, which cumulatively generate a persistent sleep debt that is difficult to repay (29).

Consistent with H1, our study demonstrated that workload was significantly and positively correlated with poor sleep quality in nurses, which is consistent with the findings of previous research (30, 31). According to the Stress–Strain Model, prolonged exposure to stressors coupled with insufficient recovery leads to the progressive accumulation and exacerbation of strain, which gives rise to long-term health impairments (13). As a well-established occupational stressor, workload exerts its deleterious effects on sleep through neuroendocrine pathways (23). Specifically, sustained high workload provokes persistent hypothalamic–pituitary–adrenal axis stimulation, which drives cortisol secretion to supraphysiological levels. The nighttime cortisol trough is essential for sleep initiation. However, chronic stress abolishes this trough, sustaining nocturnal alertness and compromising the depth and continuity of sleep (32). In addition, heavy workload exhausts individuals’ mental and physical resources, yet adequate rest and recovery are indispensable for the regeneration of these resources (33). When workload is excessive, nurses remain in a state of intense physical and mental exertion throughout their shifts. Even after leaving the workplace, many find it difficult to mentally detach from work. For example, they may replay critical incidents in their minds, worry about whether they forgot to document a medication, or anticipate the challenges of the next shift. This persistent cognitive engagement with work during off-duty hours prevents the psychological and physiological unwinding necessary for restorative sleep, thereby hindering the recovery process.

Consistent with H2, our study observed that self-regulatory fatigue was significantly and positively associated with poor sleep quality in nurses. Self-regulation depends on a finite pool of psychological resources that are consumed through sustained use and require adequate rest for replenishment (34). In the nursing context, these resources are continuously depleted by the emotional labor inherent in clinical practice, as nurses must maintain empathy and professionalism when confronted with patient suffering, death, and family distress while simultaneously suppressing their own emotional responses (35). This persistent emotional regulation, compounded by high cognitive demands during shifts and the disruptive effects of night work on physiological recovery, gradually exhausts nurses’ self-regulatory capacity (36). Once self-regulatory resources are depleted, nurses become less capable in essential processes for restorative sleep, such as emotional disengagement, which predisposes them to pre-sleep anxiety and rumination, and cognitive control, which diminishes their ability to suppress work-related intrusive thoughts during bedtime, which weakens their adherence to healthy sleep hygiene practices (37). Moreover, this relationship may constitute a bidirectional vicious cycle, as poor sleep quality compromises self-regulatory function by reducing prefrontal cortical efficiency, thereby curtailing recovery opportunities (38).

### 4.1 Implications for nursing management

Our findings have several important implications for clinical nursing management. First, given the high prevalence of poor sleep quality among nurses, institutions should adopt comprehensive health promotion policies, encompassing sleep hygiene education, routine sleep health screenings, and the creation of a supportive work environment that normalizes seeking help for sleep and mental health concerns (39, 40). Moreover, age, work unit, work experience, and night shift were significantly correlated with poor sleep quality, with stronger associations in pediatric, ICU, and emergency nurses, as well as those with longer tenure or night shift schedules. Hospital administrators should consider developing stratified health support systems that address the distinct needs of different subgroups. For senior nurses with extended work experience, regular psychological check-ups and workload adjustments may be beneficial. Hospital administrators could implement task-sharing mechanisms, such as rotating high-intensity duties among team members to prevent prolonged individual exposure, or delegating administrative tasks to designated support staff, thereby reducing the cognitive burden on senior nurses. For nurses in high-stress units, strategies such as reducing patient-to-nurse ratios, implementing structured debriefing sessions after critical incidents, and ensuring protected rest periods during shifts are recommended. For those on night-shift rotations, interventions could include optimizing shift-rotation patterns, providing nap opportunities during night shifts, and offering education on circadian rhythm management. These measures, when implemented in combination, are likely to reduce the burden of poor sleep quality among nursing staff (41). Finally, given their significant associations with poor sleep quality, both workload and self-regulatory fatigue should be addressed through several supportive interventions. Immediate amelioration may be achieved through operational measures, such as workload reallocation and mandated rest breaks, whereas enduring benefits require systematic enhancement of psychological resilience via mindfulness curricula, emotion regulation modules, and continuous professional psychological support (42).

### 4.2 Limitations

Several limitations should be considered. First, the cross-sectional design limits the establishment of causal relationships between the independent variables and poor sleep quality among nurses. Second, the use of convenience sampling to recruit participants from eight tertiary hospitals in Sichuan Province may have introduced selection bias, thereby limiting the generalizability of our findings to other cultural or healthcare settings. Third, although our statistical model incorporated key sociodemographic and occupational variables, we did not collect data on other potential confounders that may influence sleep quality, such as having infants or dependents, caffeine or stimulant consumption, and commuting time to the hospital. The absence of these variables in the analysis may have led to residual confounding. Future studies should consider including these factors to provide a more comprehensive understanding of the determinants of sleep quality in nursing populations. Finally, reliance on self-report questionnaires for all variable assessments may have introduced recall and social desirability biases, potentially resulting in an inaccurate estimation of the true prevalence of poor sleep quality in nurses.

## 5 Conclusion

Our study demonstrated that poor sleep quality was highly prevalent in nurses and was significantly associated with age, work unit, work experience, night shift, workload, and self-regulatory fatigue. These findings highlight the need for targeted interventions aimed at high-risk groups, particularly those in high-stress units and those with extended work experience or night shift schedules. However, given the cross-sectional design, causality cannot be established, and longitudinal studies are needed to confirm these associations. Implementing multilevel strategies that address both organizational and individual psychological resources may effectively mitigate sleep disturbances in nurses. Furthermore, improving nurses’ sleep quality is ultimately expected to enhance patient safety by promoting sustained vigilance and reducing the risk of clinical errors.

## Data Availability

The datasets presented in this study can be found in online repositories. The names of the repository/repositories and accession number(s) can be found in the article/supplementary material.
